# Evidence for cost-effectiveness of lifestyle primary preventions for cardiovascular disease in the Asia-Pacific Region: a systematic review

**DOI:** 10.1186/s12992-014-0079-3

**Published:** 2014-11-19

**Authors:** Lainie Sutton, Anup Karan, Ajay Mahal

**Affiliations:** School of Public Health and Preventive Medicine, Monash University, Alfred Centre, 99 Commercial Road, Melbourne, VIC 3004 Australia; Indian Institute of Public Health Gandhinagar (IIPHG), Sardar Patel Institute Campus, Thaltej Ahmedabad, 380 054 India; Nuffield Department of Population Health, University of Oxford, Oxford, UK

**Keywords:** CVD, Systematic review, Cost effectiveness, Asia-pacific region

## Abstract

**Background:**

Countries of the Asia Pacific region account for a major share of the global burden of disease due to cardiovascular disease (CVD) and this burden is rising over time. Modifiable behavioural risk factors for CVD are considered a key target for reduction in incidence but their effectiveness and cost-effectiveness tend to depend on country context. However, no systematic assessment of cost-effectiveness of interventions addressing behavioural risk factors in the region exists.

**Methods:**

A systematic review of the published literature on cost-effectiveness of interventions targeting modifiable behavioural risk factors for CVD was undertaken. Inclusion criteria were (a) countries in Asia and the Pacific, (b) studies that had conducted economic evaluations of interventions (c) published papers in major economic and public health databases and (d) a comprehensive list of search words to identify appropriate articles. All authors independently examined the final list of articles relating to methodology and findings.

**Results:**

Under our inclusion criteria a total of 28 studies, with baseline years ranging from 1990 to 2012, were included in the review, 19 conducted in high-income countries of the region. Reviewed studies assessed cost-effectiveness of interventions for tobacco control, alcohol reduction, salt intake control, physical activity and dietary interventions. The majority of cost-effectiveness analyses were simulation analyses mostly relying on developed country data, and only 6 studies used effectiveness data from RCTs in the region. Other than for Australia, no direct conclusions could be drawn about cost-effectiveness of interventions targeting behavioural risk factors due to the small number of studies, interventions that varied widely in design, and varied methods for measurement of costs associated with interventions.

**Conclusions:**

Good quality cost-effectiveness information on interventions targeting behavioural interventions for the Asia-Pacific region remains a major gap in the literature.

**Electronic supplementary material:**

The online version of this article (doi:10.1186/s12992-014-0079-3) contains supplementary material, which is available to authorized users.

## Background

In 2010, deaths due to cardiovascular disease (CVD) in the Asia-Pacific region accounted for 14% of all deaths and 48% of CVD-related deaths worldwide [1]. The share of CVD-related deaths in all-cause deaths in the Asia-Pacific region has also been rising over time - from 40% in 1990 to 48% in 2010 [1,2]. The most recent figures from the Institute for Health Metrics and Evaluation in 2010 also show that countries of the Asia-Pacific Region experienced 52% of global disability-adjusted life years (DALYs) lost due to CVD (World Health Organization (WHO)) [2,3].

The growing incidence of CVD implies adverse economic consequences for countries in the region [2,4]. Thus, smoking in China was estimated to impose an economic burden of US$28.9 billion in 2008, with medical treatment costs rising by 154% and indirect costs (such as foregone productivity) by 376% between 2000 and 2008 [5]. It has also been projected that in the absence of preventive action, CVD could cost Pakistan US$31 billion and India US$237 billion by 2015 in lost GDP [6]. A recent study [7] suggests that heart disease significantly increased out of pocket health spending, reduced labour force participation rate and increased indebtedness among Indian households.

In 2010, the vast majority of CVD-related deaths in the Asia-Pacific Region were due to hypertensive and ischemic heart disease, and stroke [1]. Risk factors for these conditions can be behavioural, metabolic, (hypertension, hyperglycaemia, abnormal serum lipid levels, or overweight/ obesity), psychological or genetic [8]. Much of the research on CVD risk factors comes from high income countries, but it has been shown that the various metabolic risk factors are also rising in the Asia Pacific Region [9]. Evidence from the Framingham Heart Study, the WHO MONICA Project and elsewhere has also shown that behavioural modification can influence metabolic risk factors for CVD [10-12]. Examples of behavioural changes include reduced intake of dietary salt, smoking cessation, trans-fat being replaced by polyunsaturated fat, and increased physical activity [8].

### Why it is important to do this review

Although the causal linkages between primary prevention and CVD-related morbidity and mortality outcomes are well understood, the evidence on the feasibility and cost-effectiveness of such primary prevention interventions is less clear in the Asia-Pacific region, especially in low- and middle-income countries. Studies conducted predominantly in high-income settings suggest that behavioural lifestyle interventions aimed at lowering morbidity and mortality due to CVD are cost-effective when implemented at the population level or when targeting high risk groups [13,14]. A recent WHO report also suggests addressing behavioural risk factors due to their cost-effectiveness, and their relative ease and speed of implementation [15,16].

However, feasibility and cost-effectiveness tend to be context-specific and difficult to generalise across countries [14]. One study [17] modelled population-wide salt and tobacco reduction interventions for several countries and found large variations in government spending associated with implementation (China US$22 per capita; Vietnam US$7 per capita; and Philippines US$14 per capita). To our knowledge no other review has examined the evidence for cost-effectiveness of various CVD lifestyle primary preventions in the Asia-Pacific Region, although a review from 2012 examined pharmaceutical primary preventive interventions in low- and middle-income countries of the region [18].

Although our focus is on low- and middle-income countries of the region, we included Australia, New Zealand and Japan for three reasons. First, there are migration links between these countries and the rest of the region: nearly two-thirds of all migrants to Australia were from the Asia-Pacific region in 2012-13 and lessons from the Australian experience may apply to specific country settings [19]. Second there are institutional linkages. For instance, the World Health Organisation’s Asia Pacific Observatory on Health Systems and Policies which connects scientific and policy-making bodies throughout the region and acts as a conduit for promoting best-practice health programs throughout the region based on evidence sharing includes both Australia and New Zealand in its membership [20]. In this context, Australia’s tobacco-related policy actions could be of great relevance in much of Asia, including China and India [21]. Finally, the epidemiological profile of the population in countries of the region will increasingly resemble its more developed counterparts in the region, reflecting rising incomes and convergence in dietary patterns towards western-style foods [22]. Thus interventions that have previously been considered in the high-income countries of the region may become relevant for their low- and middle-income counterparts in future years.

## Methods

### Types of primary prevention interventions

This review was restricted to primary prevention interventions among adults as long as they targeted at least one of the main behavioural risk factors for CVD. The interventions covered included (but were not restricted to) nutritional programs, physical activity programs (individual/group/community-wide programs), food taxation, salt reduction programs, health promotion advertising, behavioural counselling (dietary, physical activity etc. versus standard care/no primary prevention).

### Types of studies

Studies were considered for evaluation if they were in the form of [i] economic evaluation studies (i.e. cost-effectiveness analyses, cost-utility analyses, cost-benefit analyses) of primary lifestyle/behavioural interventions for CVD using case-control, cohort, cross-sectional or randomised controlled trial methods; [ii] simulation studies estimating economic effectiveness for lifestyle/behavioural primary prevention interventions for CVD; [iii] studies published in English; [iv] studies that were limited to adult populations in Asia and the Pacific.

Studies were excluded if they [i] were in the form of letters, abstracts, comments, case reports, editorials, descriptive studies, ecological studies and conference papers; [ii] involved non-human subjects; [iii] were conducted outside of the Asia-Pacific Region; and [iv] did not include information on outcomes (e.g., pure costing studies), or did not include information on intervention costs (only gross economic benefits were estimated).

### Search strategy

During October 2013 we searched major information sources such as the Cochrane database, Pubmed, Scopus, Database of Promoting Health Effectiveness reviews and the website of the WHO (for the full list of sources and search strategy see Additional file 1).

### Study selection

Search results produced titles and abstract for all studies, which were then assessed against our eligibility criteria to determine inclusion for full text review. If an article was rejected, the reason was recorded; if a study was earmarked for inclusion, it was reviewed in its entirety according to an extended standardised eligibility checklist (Additional file 2).

### Data collection process and data items

Pre-determined data was extracted from each of the studies selected for inclusion in the review using a tested data extraction form (Additional file 3). This data included the year of publication, setting (country and within-country location), target population, study type, economic modelling method, intervention type, health implications, uncertainty considerations made (i.e. sensitivity analysis, discounting), economic outcomes and costs and the perspective used (health sector, societal etc.).

### Risk of bias in individual studies (quality review)

Full text articles were appraised for quality using the criteria for economic studies’ evaluation in Evers et al. [23]. Studies were ranked, with a ‘low-risk’ study (++) deemed to have the least risk of bias and considered to be a rigorous economic study (Additional file 4). The quality criteria took into account the design of the study in terms of the participants, the primary prevention and control alternative; the frequency, duration and intensity of any intervention; measurable outcomes; whether any conflicts of interest were present; and whether ethical approval was obtained. The study design was assessed in terms of the type of evaluation, the basis of this evaluation (e.g., randomised controlled trial, cross-sectional study, simulation modelling, etc.) and the perspective of the study, where a societal perspective is considered the default position. If another perspective was taken an explanation would have to be provided as to why certain costs or effects were not considered or included. If a study period exceeded one year, discounting of future costs would have to be demonstrated. The method for evaluation should have been clearly stated and all relevant outcomes shown, with sensitivity analysis. Studies were ranked according to the NICE scale from ++ to + to – from lowest to highest risk of bias [24].

### Synthesis of studies

Studies were initially classified by whether they included cost-effectiveness analyses (CEA), where cost is relative to some utility measure (e.g. DALY, QALY, LYS) or another economic evaluation approach (e.g., cost-benefit analysis). For CEA, an assessment of cost-effectiveness was then made for each study or primary prevention intervention within each study. A study was considered very cost-effective if cost per DALY saved was less than GDP per capita, cost-effective if cost per DALY was between 1 to 3 times GDP per capita and not cost-effective otherwise [25]. GDP per capita for the year in question was obtained from the World Bank Development Indicators Database [26].

## Results

### Study selection and characteristics

**Figure 1:**
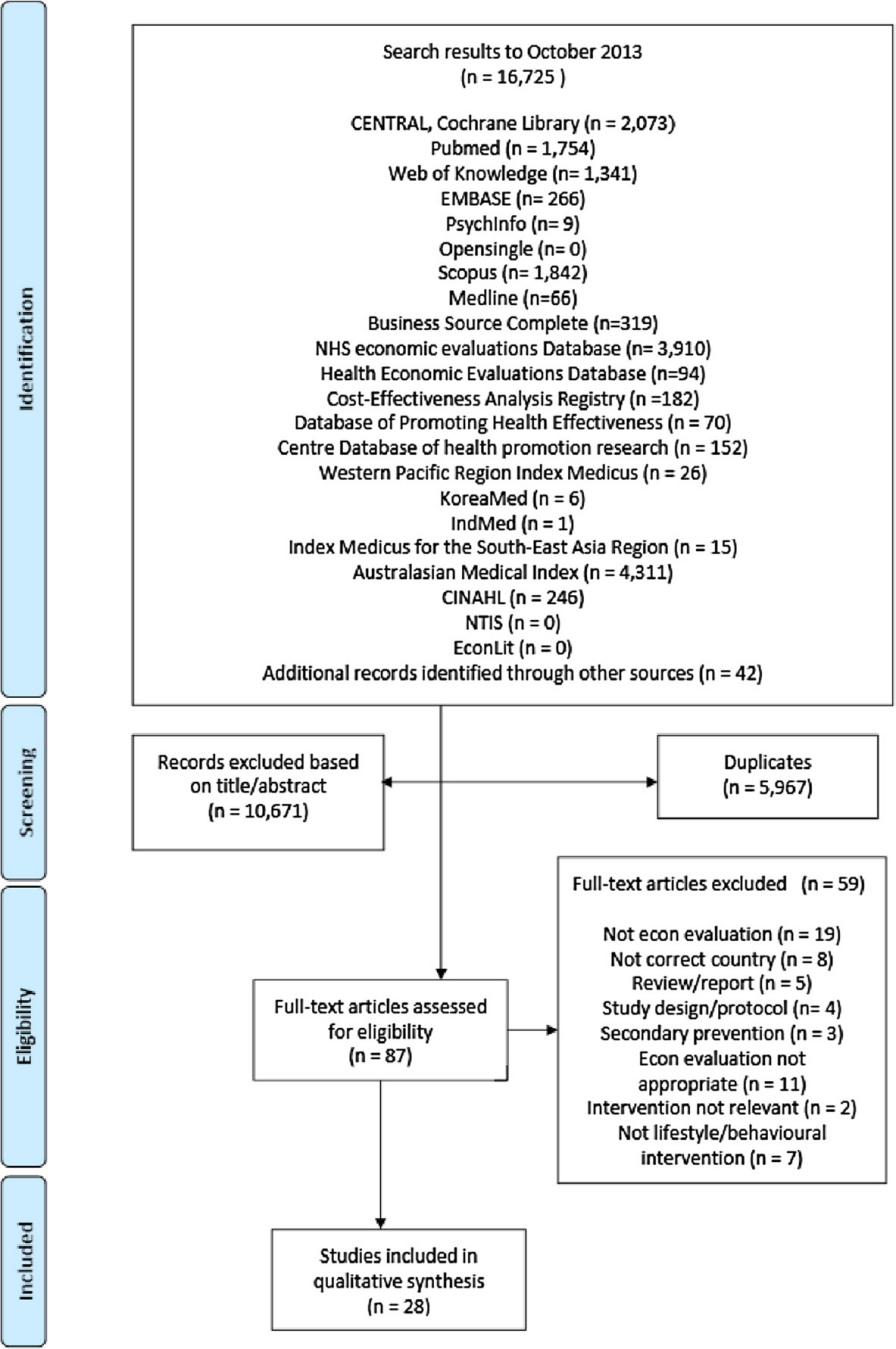
**Flow diagram of studies in the Asia Pacific Region investigating cost-effectiveness of lifestyle or behavioural interventions for cardiovascular disease primary prevention.**

The search yielded 16,725 results, of which 87 full texts were retrieved for further assessment. Subsequently, 28 were deemed suitable to be included in the qualitative review using our selection criteria. Primarily studies were excluded due to the research design being incompatible with our criteria and prevention interventions not being lifestyle or behavioural (Figure 1).

The studies retrieved for review predominantly used the period 2000-2012 as baseline years, with 4 additional studies [25,27-29] using 1990 to 1997 as baseline (the studies are summarized in Table 1).

**Table 1:** **Study characteristics: location, study design and economic perspective**

**Study**	**Country/baseline year**	**Intervention length**	**Follow-up**	**Study population**	**Key intervention components**	**Control**	**Perspective (e.g. health sector)**
**Amarasinghe** [30]	Australia, 2006	1 Year	1 Year	18 yrs + in Western Australia, male and female, CVD risk level unknown	GP advice for sufficient physical activity (150+ minutes/week) upon random presentation at clinic (6 visits)	No GP advice on physical activity	Health Sector
**Cecchini** [31]	China, India, 2008	Lifetime of population at baseline or up to 100 yrs	Lifetime	Whole population	Int1 Worksite health promotion; Int2 Compulsory food labelling; Int3 Mass media health promotion campaigns; Int4 Fiscal measures affecting fruit and vegetable and food high in fat	No intervention	Health sector
**Cobiac** [32]	Australia, 2003	Lifetime of population at baseline or up to 100 yrs	Lifetime	Whole population (15+ yrs)	Int1 Volumetric taxation; Int2 Advertising bans; Int3 Licensing controls on operating hours; Int4 Brief intervention by GP; Int 5 Brief intervention by GP with GP telemarketing and support; Int6 Residential treatment; Int7 Increase legal age; Int8 Drink driving campaigns.	Current best practice/‘do nothing’ (random breath testing present)	Health sector
**Cobiac** [33]	Australia, 2003	Lifetime of population at baseline	6 Months	Whole population (adult, BMI ≥25 kg/m^2^, don’t eat at least 7 serves of fruit and vegetables/day, don’t get 30+ minutes moderate exercise at least 5x/week)	Int1 Lighten up (group counselling for changing physical activity and nutrition patterns); Int2 Weight watchers (low-calorie diet and physical activity advice)	No intervention	Health sector
**Cobiac** [34]	Australia, 2003	Lifetime of population at baseline	Lifetime	Whole population (15+ yrs at baseline)	Int1 Pedometers; Int2 Mass media campaign; Int3 Internet advertising; Int4 GP physical activity prescription program; Int5 Travel smart program to encourage use of active transport; Int6 GP referral to exercise physiologist.	Current practice	Societal and health sectors
**Cobiac** [35]	Australia, 2003	Lifetime of population at baseline	Lifetime	Whole population (30 + yrs at baseline)	Int1 Govt. incentives for moderate reduction in salt in processed foods by manufacturers and product labelling (voluntary); Int2 Govt. mandate to moderate salt limits in processed foods; Int3 Dietary advice for those at increased risk of CVD; Int4 Dietary advice for those at high risk of CVD	No intervention	Health sector
**Cobiac** [36]	Australia, 2003	Lifetime of population at baseline	Lifetime	Whole adult population	Int1 Community-based events, sponsorship, promotion; Int2 Information mail-out (multiple re-tailored); Int3 Information mail-out (multiple tailored); Int4 Information mail-out (tailored); Int5 Individual and group dietary counselling; Int6 Individual dietary counselling; Int7 Telephone counselling and information mail-out	No intervention	Health sector
**Cobiac** [37]	Australia, 2008	Lifetime of population at baseline	Lifetime	Whole population (35-84 yrs at baseline, never experienced heart disease or stroke, all CVD risk levels)	Community: Int1 Heart health program; Int2 Mandatory reduction of salt in manufacture of bread, cereals and margarines. Individual: Int3 Dietary advice from doctor or dietician; Int4 Referral to more intensive lifestyle program with specialised counselling (≥15% risk of CVD); Int5 Advice from doctor to switch to phytosterol enriched margarine (≥15% risk of CVD)	Current best practice (‘do nothing’)	Health sector
**Dalziel** [38]	Australia, 2003	6 Months – 6 Years	1 session –1 Year	Unclear – clinical trials were in adults	8 dietary interventions	No intervention	Societal perspective
**Dalziel** [39]	New Zealand, 2000	Lifetime (40 years)	1 Year	Adults 40-79 yrs, M&F, not getting 2.5 hrs physical activity per week (n = 878)	Physical activity counselling program (verbal advice and written exercise program by GP or Nurse and telephone exercise specialist follow-up)	Best practice	Health Sector
**Elley** [40]	New Zealand, 2000	1 Year	3 Months	Adults 40-79 yrs, M&F, not getting 2.5 hrs physical activity per week (n = 878)	Physical activity counselling program (verbal advice and written exercise program by GP or Nurse and telephone exercise specialist follow-up)	Best practice	Programme funder Perspective
**Forster** [41]	Australia, 2003	Lifetime of population at baseline or 100 yrs	6-12 Months	Adults 20 yrs + overweight and obese	Int1:Hypertension diet with exercise; Int2:Low-fat diet	No intervention	Health sector
**Ha** [42]	Vietnam, 2007	Lifetime of population at baseline or up to 100 yrs	10 Years	Adult population 30 + years at baseline, all risk levels	Int1: Reduction in salt intake through voluntary manufacturer limits, mass media campaign; Int2:Mass media campaign to reduce cholesterol; Int3:Mass media campaign to reduce tobacco; Int4:Interventions 1-3 combined	No intervention	Health sector
**Higashi** [43]	Vietnam, 2006	Lifetime of population at baseline	1 Session	Adult population 15 + y at baseline, stratified by i) never smoker, ii) current smoker, iii) ex-smoker	Brief physician advice (GP or other health professional) on tobacco cessation (1 min screening and 8 min advice session)	Pharmaceutical intervention (NRT patch, NRT gum, Bupropion, Varenicline)	Health sector
**Higashi** [44]	Vietnam, 2006	Lifetime of population at baseline	1-10 Years	Adult population 15 + yr at baseline, all risk levels	Int1: Excise tax increase (55-65%); Int2: Excise tax increase (55-75%); Int3: Excise tax increase (55-85%); Int4: Graphic warning labels on cigarette packs; Int5: Mass media campaigns; Int6: Smoking bans in public; Int7: Smoking bans in workplace. All enforced for 10 years.	No intervention	Government perspective (including initial investment in interventions)
**Jafar** [45]	Pakistan, 2007	2 years	2 Years	Adult male and females 40 + years, hypertensive	Home health education (HHE) and training of GPs on BP control - Int1 :HHE + trained GP; Int2 :HHE; Int3 : Trained GP	Current practice	Societal
**Joo** [46]	South Korea, 2007	12 weeks	12 Weeks	Adults 20-64 yrs, BMI ≥ 25 kg/m^2^, waist circumference >90 cm men, 85 cm women, 30 min exercise 4 times/week	Protein-rich oriental diet and either of:- Int1: Public health centre behavioural program; Int2 : Remote behavioural program (internet, SMS)	None	Program funder perspective
**Murray** [47]	South East Asia Region (Indonesia, Sri Lanka, Thailand, Bangladesh, Bhutan, Dem Peop. Rep. Korea, India, Maldives, Myanmar, Nepal), 2000	Lifetime of population at baseline or 100 years	Lifetime	Whole population	Population interventions for BP and cholesterol control: Int1:Voluntary agreements on salt content with manufacturers; Int2:Legislated salt limits in manufactured food; Int3:Mass media campaign; Int4 : Int2 + Int3	Current practice	Government (implementation and health sector costs)
**Navarro** [48]	Australia, 2005	1 year	1 Session	Adults 18 + yrs at baseline from 10 rural communities in NSW, stratified by drinking behaviour	GP screening and brief intervention (1 session)	Current best practice	Health sector
**Oldenburg** [25]	Australia, 1990	1 year	1 Year	Adult male and female ambulance officers and paramedics	Int1:Health risk assessment (4x over 12 months); Int2 : Risk factor education (4x over 12 months plus reading material); Int3 : Behavioural counselling (risk factor education plus 1 session behavioural counselling); Int 4:Behavioural counselling plus financial incentives	No intervention	Program funder perspective
**Ortegon** [49]	South East Asia Region (Bangladesh, Bhutan, Dem. People’s Republic of Korea, India, Maldives, Myanmar, Nepal), 2005	Lifetime of population at baseline or up to 100 yrs	10 Years	Whole population 15 yrs+	Interventions implemented for 10 years	No Intervention	Program funder and health sector
					Int1: Taxes on tobacco (current excise taxation of 40%); Int2:Raise taxes on tobacco (increased excise taxation to 60%); Int3:Enforce bans on tobacco advertising; Int4:Clean indoor air in public places through legislation and enforcement; Int5:Warning labels on cigarette packs; Int6:Brief advice to help quit; Int7:Counselling to help quit; Int8: Voluntary reduction in salt in industry (15%); Int9: Legislated reduction in salt in industry (30%); Int10:Mass media education on BMI and cholesterol		
**Pritchard** [28]	Australia, 1992	1 year	1 Year	25-65 yr old men and women with one or more of: overweight, hypertension, type 2 Diabetes mellitus	6 sessions of counselling on good nutrition and exercise by: Int1: Doctor/dietician; Int2: Dietician only	No counselling	Program funder
**Ranson** [29]	East Asia & Pacific, South Asia, 1995	Lifetime of participants at baseline	Lifetime	Smokers 15 + years	Public policy control interventions: Int1: 10% price increase; Int2 :Non-price increase, non-pharmaceutical (e.g. mass media)	‘Do nothing’	Program funder (public sector)
**Sacks** [50]	Australia, 2003	Lifetime of participants at baseline	Lifetime	Whole population ≥20 years at baseline	Int1: Traffic light labelling of food based on nutritional content; Int2 :Junk food tax (10% rise in prices for consumers)	No intervention	Health sector (with some industry costs included)
**Salkeld** [27]	Australia, 1990	Lifetime of participants at baseline	1 Year	Male and female, selected by GP for at risk of CVD	Int1:Video intervention for lifestyle behaviours (n = 270); Int2:Video + self-help booklet (n = 232)	Routine care (n = 255)	Health sector (govt.)
**Shearer** [51]	Australia, 2003	6 months	6 Months	Whole adult population, smokers	Int1:Brief advice by health professional (2x 10 min visits); Int2:Telephone counselling (4x 10 min calls)	No intervention	Program funder (govt.)
**Snowdon** [52]	Fiji, Tonga, 2006	Lifetime	Lifetime	Whole population	Policy changes around food price, storage, manufacture, items available for consumption	‘Do nothing’	Govt. (cost offsets excluded)
**Zomer** [53]	Australia, 2012	10 years or death of baseline population	10 Years	10,000 adults ≥25 yrs with hypertension and metabolic syndrome, no CVD history (based on subsection of AusDiab Study participants)	Daily consumption of dark chocolate (500-1000 mg/day)	No dark chocolate consumption	Health sector

*Abbreviations*: *Int* Intervention, *CVD* Cardiovascular Disease, *GP* General Practitioner, *NRT* Nicotine Replacement Therapy, *HHE* Home Health Education, *BMI* Body Mass Index, *Govt* Government.

Almost two-thirds of the studies were located within high-income countries of the region (19/28), with 16 in Australia. A large number (22/28) were simulation analyses, while 6 cost-effectiveness assessments were embedded within randomised controlled trial prevention studies [25,27,28,40,45,46]. Only 2 studies [28,46] did not include some type of sensitivity analysis for their findings. 18 of the studies were population-based and most studies (23/28) discussed the generalizability of their findings for other populations. Many studies took a government perspective (including costs of the intervention and healthcare cost offsets), with 4 taking a perspective that included the associated household economic burden [33,34,38,45]. 19 of the 28 studies included DALYs or QALYs as outcomes, based either on CVD outcomes alone, or on CVD outcomes plus cancers and diabetes (and COPD in some cases), for cost-effectiveness analyses. Outcomes in other studies included indicators of changes in risk factors for CVD (e.g., weight reduction, number of smokers quitting, physical activity and systolic BP), and deaths averted.

Given the focus of primary prevention interventions on lifestyle behaviours, it was expected that authors take into account programme compliance rates of participants and their decay over time [54]. Many studies considered this aspect in the analysis, yet 7/28 did not provide any estimate of the effect of decay [28,37,45-48,52]. Finally, with a few exceptions [28,29,43,49] that did not itemize costs, most included studies used appropriate costing for interventions and other peripheries.

### Risk of bias across studies

Table 2 reports our findings on the risk of bias across the included studies.

**Table 2:** **Assessing risk of bias in studies included in the review**

**Study**	**Sensitivity analysis; discount rate (per annum); perspective (e.g. health sector)**	**Measurement of costs (physical units, valued appropriately, all costs included, itemised)**	**QALY/DALY weights clearly defined**	**Appropriate conclusions based on results**	**Generalisability of study to other settings/ patient groups discussed**	**Conflict/s of interest disclaimed**	**Ethical issues discussed**	**Risk analysis (++ low risk, + moderate risk, - high risk)**
**Amarasinghe** [30]	Uncertainty of compliance and subsidy rates (10, 20, 25, 50, 75, 100%); N/A; Health Sector	✓	✓	✓	✗	✓	✗	**+**
**Cecchini** [31]	Simplified (sd +15% of mean, max and min + -60% mean); 3% (costs and effects); Health Sector	✗	✗	✓	✓	✓	✗	**-**
**Cobiac** [32]	Monte Carlo (2000 iterations) 95% CI; 3% (costs and effects); Health Sector	✗	✓	✓	✗	✓	✗	**-**
**Cobiac** [33]	Monte Carlo, 95% CI; 3% (costs and effects); Health Sector	✗	✓	✓	✗	✓	✗	**-**
**Cobiac** [34]	Monte Carlo (2000 iterations), 95% CI; 3% (costs and effects); Societal and health sectors	✓	✓	✓	✗	✓	✗	**+**
**Cobiac** [35]	Monte Carlo, 95% CI; 3% (costs and effects); Health Sector	✓	✓	✓	✗	✓	✗	**+**
**Cobiac** [36]	Monte Carlo, 95% CI; 3% (costs and effects); Health Sector	✓	✓	✓	✓	✓	✗	**++**
**Cobiac** [37]	Monte Carlo, 95% CI; 3% (costs and effects); Health Sector	✓	✓	✓	✓	✓	✓	**++**
**Dalziel** [38]	Uni-variate (effect size, cost, utility, time horizon); 5% (future costs): Societal	✗	✓	✓	✓	✗	✓	**+**
**Dalziel** [39]	One-way (1000 simulations); 5% (future costs); Health Sector	✗	✓	✓	✓	✗	✗	**++**
**Elley** [40]	Least squares regression model, 95% CI; 5% (costs); Programme Funder	✗	N/A	✗	✓	✗	✓	**-**
**Forster** [41]	Monte Carlo (2000 iterations), 95% UI; 3% (costs and effects); Health Sector	✓	✓	✓	✓✓	✓	✗	**++**
**Ha** [42]	Monte Carlo (1000 iterations); 3%; Health Sector	✗	✗	✓	✓	✓	✗	**-**
**Higashi** [43]	Monte Carlo (2000 iterations), 95% CI; 3% (costs and effects); Health Sector	✗	✗	✓	✓	✓	✗	**-**
**Higashi** [44]	Monte Carlo (2000 iterations), 95% UI; 3% (costs and effects); Government (implementation and maintenance)	✓	✗	✓	✓	✓	✗	**+**
**Jafar** [45]	Bayesian sensitivity (1000 repetitions), 95% CI; 5% (costs and effects); Societal	✓	✗	✗	✓	✓	✓	**+**
**Joo** [46]	No; N/A; Program Funder	✓	N/A	✗	✓	✗	✓	**+**
**Murray** [47]	Monte Carlo, multivariate uncertainty analysis (range unclear); 3% (costs and effects); Government (implementation and health sector costs)	✗	✗	✓	✓	✓	✗	**-**
**Navarro** [48]	One-way (sensitivity analysis range 39-59%); N/A; Health Sector	✓	N/A	✓	✓	✓	✗	**++**
**Oldenburg** [25]	Multivariate; N/A; Program Funder	✗	N/A	✓	✓	✗	✓	**+**
**Ortegon** [49]	One way, probabilistic uncertainty analysis; 3% (costs and effects); Program Funder and Health Sector	✗	✓	✗	✓	✓	✗	**-**
**Pritchard** [28]	No; N/A; Program Funder	✗	N/A	✓	✓	✓	✓	**++**
**Ranson** [29]	Uncertainty around discount rates; 3% (costs and effects); Program Funder (public sector)	✗	✓	✓	✓	✓	✗	**+**
**Sacks** [50]	Monte Carlo (2000 iterations), 95% CI; 3% (costs and effects); Health Sector (with some industry costs included)	✗	✗	✓	✓	✓	✗	**-**
**Salkeld** [27]	One-way; 5% (costs and effects); Health Sector (govt)	✓	✓	✓	✓	✗	✓	**++**
**Shearer** [51]	Multivariate (effectiveness, resource use and costs); N/A; Program Funder (govt.)	✗	N/A	✓	✓	✗	✗	**-**
**Snowdon** [52]	Monte Carlo (5000 iterations per country model); probabilistic uncertainty analysis of deaths averted; N/A: Govt. (cost offsets excluded)	✗	N/A	✗	✓	✗	✗	**-**
**Zomer** [53]	Monte Carlo (1000 iterations) interquartile range; Uncertainty based on compliance levels; 5% (costs and effects): Health Sector	✗	✗	✗	✓	✓	✗	**-**

*Abbreviations*: *Govt* Government, *CI* Confidence Interval, *UI* Uncertainty Interval, *sd* Standard Deviation.

Only one study [53] used industry funding but it was specified that there was no conflict of interest with the funding body. 8 of the 28 studies had a potential for conflict of interest based on the lack of a disclaimer, a vague disclaimer or the possibility of stakeholder involvement in the analyses [25,27,38-40,46,51,52]. We also compared estimates of primary prevention effectiveness when there was some conflict of interest in the study with estimates of primary prevention effectiveness when studies were deemed to have no conflict of interest. Studies which had some risk of conflict of interest demonstrated a positive effect of the intervention (cost-effective to very cost-effective outcome) about 70 percent of the time, whereas 80 percent of the studies deemed to be without a conflict of interest showed a positive effect of the intervention. Most studies’ conclusions followed the results provided, with a few exceptions providing incomplete or selective conclusions relative to the results observed. Overall we deemed 7/28 studies to be at low risk of bias [27,28,36,37,39,41,48] (with all of these studies conducted in Australia), 12/28 studies to be high risk [31-33,40,42,43,47,49-53] and the remainder (9/28) as having a moderate risk of bias.

### Evidence for cost-effectiveness of primary prevention

Many studies evaluated multiple primary prevention interventions, and some targeted multiple risk factors. For example Jafar et al. [45] assessed 3 interventions for hypertensive patients: a home-based health education programme delivered by community health workers, a programme delivered by general practitioners, and a combination of the two, compared to no intervention. The interventions targeted diets, physical activity and smoking, and the primary outcomes were systolic blood pressure reduction and DALYs saved. Thus, some studies appear under multiple heads when assessing the impacts of interventions. Table 3 summarizes our main findings on economic evaluation.

**Table 3:** **Cost-effectiveness of interventions**

**Study**	**Currency**	**Costs**	**ICER (Incremental cost-effectiveness ratio)**	**Cost-effectiveness**
**Amarasinghe** [30]	2003 AUD	$16 million saved on IHD treatment	When 100% compliance, $20 subsidy per GP visit $810/DALY averted (all causes)	Very cost-effective
		$18 million saved on stroke treatment	When 50% compliance, $25 subsidy rate, $11,000/DALY averted (all causes)	
**Cecchini** [31]	2005 US$		At 20 yrs, compared to control – Int1 China $7785/DALY averted, India $6151/DALY averted; Int2 China $71/DALY averted, India $952/DALY averted; Int3 China $7188/DALY averted, India $15552/DALY averted; Int4 China cost-saving, India cost-saving	Int 1 Cost-effective in China, not cost-effective in India; Int 2 very cost-effective in China, cost-effective in India; Int 3 very cost-effective in China, not cost-effective in India; Int4 very cost-effective
**Cobiac** [32]	2003 AUD		Compared with current best practice:	Int1-5, 7-8 very cost-effective; Int 6 not cost-effective
			Int1 Dominant; Int2 Dominant; Int3 $3300/DALY averted; Int4 $6800/DALY averted; Int5 $10,000/DALY averted; Int6 $190,000/DALY averted; Int7 Dominant; Int8 $14000/DALY averted	
**Cobiac** [33]	2003 AUD		Compared to ‘do nothing’	Cost-effective
			Int1 $130,000/DALY averted; Int2 $140,000/DALY averted	
**Cobiac** [34]	2003 AUD		Compared to ‘do nothing’ (medians)	Int 1-5 very cost-effective; Int 6 cost-effective
			Int1 Dominant; Int2 Dominant; Int3 $3000/DALY averted; Int4 $11,000/DALY averted; Int5 $18,000/DALY averted; Int6 $79000/DALY averted	
**Cobiac** [35]	2003 AUD		Compared to ‘do nothing’ :	Int1 and 2 cost-effective; Int 3 not cost-effective; Int4 unlikely to be cost-effective
			Int1 Dominant; Int2 Dominant; Int3 $260000-$390000/DALY averted; Int4 160000-250000/DALY averted	
**Cobiac** [36]	2003 AUD		Compared to ‘do nothing’, at 1 yr (assumed 50% decay in effectiveness after implementation):	Int 1-4 very cost-effective; Int 5 and 6 not cost-effective; Int7 cost-effective
			Int1 Dominant; Int2 $8600/DALY averted; Int3 $12000/DALY averted; Int4 $27000/DALY averted; Int5 $280000/DALY averted; Int6 $950000/DALY averted; Int7 $84000/DALY averted	
**Cobiac** [37]	2008 AUD		Compared to ‘do nothing’:	Int1 and 2 very cost-effective; Int 3 and 4 cost-effective; Int 5 not cost-effective
			Int1 Dominant (Dominant to Dominant); Int2 $44000/DALY averted ($19000-$100000/DALY averted); Int3 $1000000/DALY averted ($610000-2400000/DALY averted); Int4 $1400000/DALY averted ($960000-2500000/DALY averted); Int5 $3200000/DALY averted ($1900000-5900000/DALY averted)	
**Dalziel** [38]	2003 AUD		Compared to control: $46/QALY to $19800/QALY for all 8 interventions	Very cost-effective
**Dalziel** [39]	2001 NZ$		Compared to control, at 40 year time point:	Very cost-effective
			For intervention implemented 1 year, effects lasting 4 years: $2053/QALY gained; Effects lasting 5 years: $1663/QALY gained; Effects lasting 10 years: $1160/QALY gained	
**Elley** [40]	2001 NZ$		At 12 months, compared to control: $1756 for 1 adult to move from a sedentary to active state; Program cost $170.45 per patient per year	Inconclusive
**Forster** [41]	2003 AUD		Compared to control: Int 1 $12000/DALY averted (cost saving -$68000/DALY averted); Int 2 $13000/DALY averted (cost saving -$130000/DALY averted)	Very cost-effective
**Ha** [42]	2007 VND	Int1 1945002/DALY averted; Int2 12324059/DALY averted; Int3 2416075/DALY averted; Int4 2211140/DALY	Compared to control: All interventions Dominated	Very cost-effective
**Higashi** [43]	2006 VND	1742000/DALY averted (I$ 543/DALY averted) physician advice	Compared to control: All pharmaceuticals dominated by physician advice.	Physician advice very cost-effective; pharmaceutical interventions not cost-effective
**Higashi** [44]	2006 VND		Compared to control: All interventions dominate	Very cost-effective
			Int1 8600VND/DALY averted (3400, 20100);Int2 4200VND/DALY averted (1700, 9900); Int3 2900VND/DALY averted (1100, 6700); Int4 500VND/DALY averted (300,1200); Int5 78300VND/DALY averted (43700, 176300); Int6 67900VND/DALY averted (28200-332000); Int7 336800VND/DALY averted (169300, 822900)	
**Jafar** [45]	2007 US$		Compared to no intervention: Int 1$23/mmHg ($7-$101/mmHg); Int 2 Dominated (dominated to 730/mmHg); Int 3 $206/mmHg (Dominated to $807/mmHg)	Inconclusive
**Joo** [46]	2007 US$	Public health int $976/person to reach target weight; Remote int. $1637/person to reach target weight		Inconclusive
**Murray** [47]	I$ 2000	Int 1 $37/DALY averted; Int2$19/DALY averted; Int3 $14/DALY averted; Int4 $17/DALY averted (personal interventions $36-90/DALY)	Int 2 compared to Int3 $14/DALY averted; (Int 2 to Int 4) compared to (Int 3 to Int4) $20/DALY averted	Very cost-effective
**Navarro** [48]	2005-2006 AUD		Compared to ‘do nothing’: 10% increase in screening rate $217/risky drinker reducing alcohol consumption; 20% increase $205; 100% increase $216	Inconclusive
**Oldenburg** [25]	1990 AUD	Int3 (only intervention which reached the maintenance stage of behavioural intervention) $22.06/unit of CVD risk reduction		Inconclusive
**Ortegon** [49]	I$ 2005		Compared to no intervention: Int1 $116/DALY averted; Int2 $87/DALY averted; Int3 $187/DALY averted; Int4 $162/DALY averted; Int5 $195/DALY averted; Int6 $958/DALY averted; Int7 $1179/DALY averted; Int8 $197/DALY averted; Int9 $901991/DALY averted; Int10 $191/DALY averted	Int 1-5, 8, 10 very cost-effective; Int6 not cost-effective in Myanmar, cost-effective in Bangladesh, Dem Rep Korea, India, very cost-effective Bhutan, Maldives; Int7 not cost-effective in Myanmar or Nepal, cost-effective in Bangladesh, India, Dem Rep Korea, very cost-effective in Bhutan and Maldives; Int9 not cost-effective
**Pritchard** [28]	1993-4 AUD		Compared to control: Int 1 $9.76/extra kg lost (12% reduction in BP); Int 2 $7.30/extra kg lost (7% reduction in BP)	Inconclusive
**Ranson** [29]	1997 USD		Compared to control: Int 1: East Asia & Pacific $2-50/DALY averted, South Asia $1-33/DALY averted; Int 2: East Asia & Pacific $25-510/DALY averted, South Asia $16-326/DALY averted	Int 1 Very cost-effective; Int 2 cost-effective to very cost-effective
**Sacks** [50]	2003 AUD		Compared to ‘do nothing’: Int 1 Dominant ($30/DALY averted, 95% CI 20-40); Int 2 Dominant ($1800/DALY averted, 95% CI 1360-2170)	Very cost-effective
**Salkeld** [27]	1994 AUD		Compared to routine care, both interventions not cost-effective or effective (no significant change in risk); Except for Int 1 for high risk males $39440/LYS and $29574/QALY	Int 1 for high risk males very cost-effective
**Shearer** [51]	2003 AUD	Brief advice: $1910/quitter ($1273-3820); Telephone counselling: $606/quitter ($505-757)		Inconclusive
**Snowdon** [52]	2006 Fiji dollar (FJD)	At 1 year Most effective: Tonga – Ban on sale of all fatty meats TOP 30974/6.61 deaths averted; Fiji – cool storage available at all markets		Inconclusive
	2006 Tongan Pa’anga (TOP)	FJD1600149/65.54 deaths averted; Lowest costs: Tonga – removal of licensing requirements for roadside vendors selling local produce TOP0/death averted; Fiji- import duty (15%) added to all oils FJD396/17.43 deaths averted		
**Zomer** [53]	2012 AUD	100% compliance: $50,000/LYS		Very cost-effective

NB. All studies are cost-effectiveness analyses. Abbreviations: CEA – Cost-Effectiveness Analysis; FJD – Fijian Dollar; TOP – Tongan Pa-anga; AUD – Australian Dollar; DALY – Disability-Adjusted Life Years; QALY – Quality-Adjusted Life Years; LYS – Life Years Saved; CI – Confidence Interval; USD – United States Dollar; I$ - international dollars; VND – Vietnamese Dong; mmHg – millimetres of mercury; NZ$ - New Zealand Dollar; Int – Intervention; yrs – years; ICER – Incremental Cost-Effectiveness Ratio.

### Tobacco control

7 studies dealt with tobacco control, of which 5 were in low- and middle-income countries [42-45,49], 1 in a high-income country [51], and one in countries of all income levels [29]. Two studies [45,51] did not demonstrate cost-effectiveness because their outcomes were other than QALYs or DALYs, namely the numbers of individuals who quit smoking and reduction in systolic BP [45,51]. 3 studies demonstrated interventions that were very cost-effective [42-44] with [43] investigating the cost-effectiveness of brief physician advice (general practitioner [GP] or other health professional) on tobacco cessation (1 min screening and 8 min advice session); [44] analysing excise tax increases (55%-65%, 55%-75%, 55%-85%), graphic warning labels on cigarette packs, mass media campaigns, smoking bans in public and smoking bans in the workplace; and [42] looking at a mass media campaign to reduce tobacco use. 2 studies assessed interventions that ranged from not cost-effective to very cost-effective depending on location and intervention type [29,49]. In study [29], compared to ‘do nothing’, a 10% price increase (55%-65%) in cigarettes was very cost-effective, and mass media interventions ranged from cost-effective to very cost-effective (from $25-510/DALY averted in East Asia & Pacific and $16-326/DALY averted in South Asia). In study [49] which used simulation modelling, increasing excise taxes on tobacco, bans on tobacco advertising, legislation and enforcement for clean indoor air in public places, and warning labels on cigarette packs were all very cost-effective in South and Southeast Asia.

### Alcohol reduction

We found only 2 studies that evaluated cost-effectiveness of alcohol reduction primary prevention interventions [32,48], both in Australia. Study [32] reported results ranging from not cost-effective (residential treatment for alcohol reduction) to very cost-effective (volumetric taxation, advertising bans, licensing controls on operating hours, brief primary prevention by GP, brief primary prevention by GP with telemarketing and support for GP recruitment to program, increased legal age of alcohol consumption from 18 to 21 years and mass media drink driving campaign). This study considered healthcare cost offsets in assessing cost-effectiveness. Study [48] was a cost-effectiveness analysis of an intervention consisting of screening by a GP and brief intervention if necessary (one appointment) among high-risk drinkers in a rural community in Australia. Outcomes were a reduction in high-risk drinking and the study estimated costs ranging from $175-$300 per high risk drinker moving to a low-risk status. However, no DALY or QALY outcomes were reported so a direct assessment in terms of the criteria of cost-effectiveness used in this review was not possible.

### Salt intake control

We found 4 studies on salt control primary prevention interventions (Table 1), 2 in Australia [35,37], one in Vietnam [42], and one in a set of South East Asian countries [49]. In 2 studies, interventions ranged from being not cost-effective to cost-effective/very cost-effective depending on intervention type or setting [35,49], and 2 studies [37,42] found the interventions to be very cost-effective. In study [35] incentives from government for moderate reduction in salt in processed foods and product labelling by manufacturers, and a government mandate for manufacturers to moderate salt limits in processed foods were cost-effective relative to ‘doing nothing’; and individual dietary advice for those at increased/high risk of CVD was not cost-effective. In study [49], voluntary reduction in salt by the industry (15%) was found to be very cost-effective; and a legislated 30% reduction in salt in industry also ranged from cost-effective to very cost-effective, relative to doing nothing. In [37], compared to ‘doing nothing’, the mandatory reduction of salt in the manufacture of bread, cereals and margarines was found to be very cost-effective. In [42], a reduction in salt intake through voluntary manufacturer limits and a mass media campaign were very cost-effective.

### Physical activity/diet control

Roughly three-fourths of the studies (20/28) examined diet and or physical activity-based primary preventions. Of these, 5 [31,42,45,47,52] were in low- and middle-income countries. Of the 15 studies in high-income countries, 12 studies were conducted in Australia. The most common form of prevention evaluated was counselling (18 studies), although a few studies also evaluated legislation, taxation and mass media-based interventions. All 6 randomised controlled-trial (RCT)-embedded studies included in this review were in the physical activity/diet category, and of these only 1 was conducted in a middle-income country, Pakistan [45].

The studies in low- and middle-income countries covered a variety of primary prevention interventions. A study in Fiji and Tonga [52] examined the impact of tax interventions (lowering import duties on fruits and vegetables, raising import duties on fatty oils, removal of import duty benefits for processed meats), legislation (ban sales of fatty meats and fatty processed foods) and enhancing cool storage for fruits, vegetables and fish, concluding that tax policies and legislation were likely to be much more cost-effective (in terms of deaths averted) than expansion of storage facilities. However, outcomes were not expressed in DALYs or QALYs. The study for Pakistan [45] was also difficult to assess from a cost-effectiveness perspective as the outcome was systolic BP. Study [48] used mathematical modelling together with effectiveness data from mostly high-income countries to assess the cost-effectiveness of 7 programmes directed to diets and physical activity in 6 middle-income countries, of which 2 (China and India) were in the Asia-Pacific region. The programs evaluated were school-based primary preventions, worksite primary preventions, mass media campaigns, tax policies (affecting prices for fruits, vegetables and foods rich in fat), GP counselling, regulation of food advertising and food labelling. Regulation of food advertising, food labelling and tax policies ranged from cost-effective to very cost-effective in both China and India. In China, moreover, worksite primary preventive interventions and mass media campaigns were assessed to be very cost-effective over a long (50-year) horizon. However, other interventions were not cost-effective in either country. In Vietnam, a mass media campaign aimed at reducing cholesterol [42] was assessed to be very cost-effective, although other than salt intake it was not clear if any other lifestyle factors were targeted by the intervention. A mass media campaign targeting BP and cholesterol control was also found to be very cost-effective in [47] in Southeast Asian countries.

In studies conducted in high income countries (Australia, New Zealand and South Korea), 5 either did not explicitly assess cost-effectiveness or used outcome measures that were not expressed in DALYs or QALYs [25,28,40,46,53]. Study [46], set in Korea, concluded that it was more cost-effective to educate individuals about dietary and lifestyle changes to lower weight than to do so remotely via the internet; study [40] set in New Zealand concluded that GP advice and prescription for physical activity with telephone follow-up by an exercise specialist increased physical activity among 40-79 year olds at a ‘reasonable cost’; study [28] set in Australia concluded that nutritional counselling to overweight or hypertensive patients lowered weight at a rate of $7-$10 of programme costs per lost kilogram; another study [25] concluded that behavioural counselling was more cost-effective in lowering CVD risk factors than strategies focused on risk-factor education among individuals or behavioural counselling plus financial incentives; and study [53] also set in Australia, found an intervention promoting dark chocolate consumption was very cost-effective.

The remaining 10 studies were set in Australia (1 in New Zealand). Of these, a significant number focused on counselling, with and without GPs, and these interventions were generally cost-effective. In study [30], GP advice for sufficient physical activity upon presentation at a clinic was compared to no advice and found to be cost-effective/very cost-effective depending on patient compliance rates. In study [27] GPs were provided with a video and an instructional guide to assess risk factors and to plan risk factor behaviour change and patients also received a video on lifestyle behaviours and risk factor assessment. In addition, a small group received a self-help booklet. Video-based interventions were cost-effective or very cost-effective for high-risk males, but were not cost-effective for other population subsets. In study [37], dietary advice from doctor or dietician or referral to more intensive lifestyle program with specialised counselling (for those with ≥15% risk of CVD) were found to be cost-effective compared to doing nothing. In study [39] a physical activity counselling program in New Zealand implemented by a GP or nurse with telephone follow-up was found to be very cost-effective compared to existing practice. In study [33], group counselling for changing physical activity levels and nutritional patterns, and a low-calorie diet with physical activity advice were found to be cost-effective compared to no intervention although health gains were overall quite low. On the other hand [34] found GP physical activity prescription programs to be very cost-effective and GP referral to an exercise physiologist to be cost-effective. Only one study [36] found individual and group counselling to be not cost-effective. Study [36] also assessed community-based primary prevention including sponsorship and promotion for fruit and vegetable consumption, information mail outs, all of which were very cost-effective.

Among other primary prevention interventions, study [50] found junk food taxes and food labelling targeted at Australia’s adult population to be individually very cost-effective. Study [34] assessed interventions for increasing physical activity, finding the use of pedometers, a mass media campaign, internet advertising and a program to encourage the use of active transport to be very cost-effective; study [38] examined 8 diet programs and found all to be very cost-effective; and finally, in study [41], a hypertension diet combined with exercise and a low-fat diet were compared to no intervention and both were very cost-effective, although the overall health benefits were small.

Several of these studies addressed BMI as a cardiovascular risk factor. Despite BMI by itself not being a reliable predictor of CVD, in the absence of other measures such as systolic BP and diabetes, it offers some indication that other risk factors for CVD may be present, as determined in a recent, comprehensive Lancet study [55]. Weight loss studies included in this review follow this line with weight gain considered an intermediate risk factor for CVD with studies [27,33,34,37,39,41,52] modelling QALYs or DALYs based on, for example, the relative risk of cardiovascular (and other) disease when at a particular weight. Studies [28,40,46] use weight loss as the direct outcome.

## Discussion

We find first that multiple tobacco control interventions, particularly taxes on tobacco, advertising restrictions, warning labels, mass media campaigns and GP consultations were cost-effective to very cost-effective. Second, available studies show that alcohol taxation, advertising restrictions, mass media campaigns against drunk driving and GP-based primary preventions range from cost-effective to very cost-effective, although in this case, the studies are limited to Australia. Third, voluntary manufacturer limits, or mandated or incentivized reduction in salt in processed foods were very cost-effective. However, dietary advice on salt intake for individuals at high risk of CVD was not cost-effective. Fourth, regulation of food advertising, food labelling and taxes on fatty foods/oils are very cost-effective, although the evidence on worksite primary prevention was unclear. Studies, primarily from developed countries in the region, also show that dietary and physical activity counselling through health personnel (with some follow up) is cost-effective. Community-based primary prevention on diet and physical activity were also found to be cost-effective but their benefits were small.

Unfortunately we also find a serious shortage of information on cost-effectiveness for primary lifestyle prevention related to CVD in the Asia-Pacific region. Only 28 studies met our inclusion criteria, covering various lifestyle and behavioural interventions for primary prevention of CVD. A large number of studies analysed in this review were conducted in developed countries of the region, mainly Australia. Only 8 studies were conducted exclusively in low- or middle-income countries of the Asia Pacific Region (Fiji and Tonga; China and India; Pakistan; Vietnam) so that generalizing our findings on cost-effectiveness of primary prevention interventions to the broader region is difficult. Only one study covered a broad cross-section of countries in the region, low- and high-income, and was limited to tobacco control. And a number of studies did not use DALYs or QALYs as outcomes.

Overall 20/28 of the studies demonstrated cost-effectiveness of an intervention, but we cannot rule out publication bias skewing our results towards including studies demonstrating cost-effectiveness. However, the share of studies reporting (cost) effectiveness is similar to comparable reviews in the field [56].

With the exception of [40,41] studies included in this review generally rely on short-term effects of interventions to construct cost-effectiveness estimates. Short-term effects can be misleading if there is relapse in behaviour, as in interventions targeting weight loss or smoking or, when intervention is at the population level, there are saturation effects [57]. In particular, mathematical modelling the effects of an intervention over the lifetime of an individual are susceptible to this problem given their estimates of economic impacts rely on modelling of behavioural change beyond the intervention period [27,33,38-44,49]. In addition, RCT-based analyses sometimes rely on conditions that are unlikely to be replicated in other real life settings. Sensitivity analyses can address this to an extent and many of the studies included in this review do include analyses that allow for variation in the estimates of program effectiveness. The sensitivity analyses took various forms, such as allowing for decay in behavioural interventions over time or variations in compliance rates, or in some cases, varying rates of program effectiveness. However, it is difficult to evaluate how effective these sensitivity analyses were in capturing uncertainty about the full range of outcomes that were possible and/or long-term program effects. For those studies which were modelled for over a year, discount rates were similar, with most authors discounting both costs and effects by 3 to 5 percent annually. Another limitation is that several studies did not provide a clear definition of the DALY or QALY weighting used in their analysis [31,42-45,47,50,53].

Primary prevention interventions also varied considerably in the studies considered. Interventions associated with tobacco control, alcohol reduction, physical activity/diet control and salt control can broadly be divided into counselling, legislation and mass media. Yet, in their implementation, the interventions evaluated varied by the age and occupational group of the population targeted, gender, risk level for CVD; the length of intervention; or the implementing body be it general practitioners, legislators or researchers. Thus, for counselling-mediated physical activity and diet primary prevention, the target population varied from the whole population [47], to those 20 years or older who were also overweight or obese [41] to those who were 18 years or older and ambulance officers [25].

In consequence it is difficult to aggregate the cost-effectiveness findings of individual studies, say through meta-analysis based on results from high quality studies. Much of the evidence for effectiveness, even when applied to low- and middle-income countries in the region, has come from high-income countries, some from outside the Asia-Pacific. The cost data, derived from the WHO-CHOICE model in some of the studies, conforms to a standardized approach that may not be suited to the considerable heterogeneity across health systems in the region. Moreover the assessment of costs was variable, with some studies limited to costing the primary prevention alone, others to taking account of the costs of healthcare use associated with the primary prevention and a few also including household costs.

Finally, the review makes clear the need to have graded cost-effectiveness data for multiple interventions and standardized methods for estimating costs and outcomes for individual countries in the region to guide policy choices. Australia is the only country of the Asia-Pacific Region with a developed evidence base and a relative standardisation of methods [32-37], and could serve as a benchmark for similar studies elsewhere in the region.

## Conclusion

Although there is a large international literature on the effectiveness and cost-effectiveness of primary prevention interventions targeting lifestyle factors for CVD, a closer examination points to a very limited literature in the Asia-Pacific Region. Even this limited literature is characterized by variations in the interventions evaluated across countries and use of dissimilar methodologies, and has its major focus on high-income countries in the region. This constitutes a major gap in the literature at a time when NCDs are acquiring increasing significance in the region. As countries turn towards universal healthcare coverage, policymakers will need high quality advice about efficient strategies regarding NCDs, particularly those focusing on prevention, to avoid burdening health systems with large numbers of individuals with chronic conditions that are expensive to treat.

## Additional files

Additional file 1:
**Full list of sources and search strategy.**
Additional file 2:
**Eligibility checklist.**
Additional file 3:
**Data extraction and management.**
Additional file 4:
**Assessment of risk of bias in included studies.**

## Abbreviations

CVD: Cardiovascular disease
APR: Asia-Pacific Region
DALY: Disability-adjusted life years
RR: Risk ratio
GDP: Gross domestic product
MONICA: Monitoring trends and determinants in cardiovascular disease
CEA: Cost-effectiveness analyses
QALY: Quality-adjusted life years
CE: Cost effective
VCE: Very cost-effective
NCE: Not cost effective
CI: Confidence interval
COPD: Chronic obstructive pulmonary disease
UI: Uncertainty interval
sd: Standard deviation
GP: General practitioner
SEARO: South-East Asia Regional Office
RCT: Randomised control trial
BP: Blood pressure
BMI: Body mass index
CHOICE: Choosing interventions that are cost effective
NRT: Nicotine replacement therapy
HHE: Home health education
Govt: Government
Int: Intervention
ICER: Incremental cost-effectiveness ratio
LYS: Life-years saved
FJD: Fijian dollar
TOP: Tongan pa-anga
AUD: Australian dollar
USD: United States dollar
VND: Vietnamese dong
mmHg: Millimetres of mercury
NZ$: New Zealand dollar
