# Supplementary material for: Evidence for cost-effectiveness of lifestyle primary preventions for cardiovascular disease in the Asia-Pacific Region: a systematic review
Source: Global Health. 2014 Nov 19;10:79. doi: 10.1186/s12992-014-0079-3 (PMC4251847; doi:10.1186/s12992-014-0079-3)
Supplement: Additional file 1: — Full list of sources and search strategy. [file 12992_2014_79_MOESM1_ESM.docx]

**Additional File 1**

**FULL LIST OF SOURCES AND SEARCH STRATEGY**

Sources:

Asia pacific journal of clinical nutrition (apjcn.nhri.org.tw/)

Australasian Medical index ([www.nla.gov.au/ami/](http://www.nla.gov.au/ami/))

BiblioMap – EPPI-Centre database of health promotion research (eppi.ioe.ac.uk/webdatabases/Intro.aspx?ID=7)

British Library Direct Plus ([www.bl.uk/reshelp/atyourdesk/docsupply/productsservices/bldplus](http://www.bl.uk/reshelp/atyourdesk/docsupply/productsservices/bldplus))

Business source complete (www.ebscohost.com/academic/business-source-complete)

Chinese Biomedical Literature Database (CBM) ([www.imicams.ac.cn/cbm/index.asp](http://www.imicams.ac.cn/cbm/index.asp))

CINAHL (health.ebsco.com/products/the-cinahl-database)

Cochrane CENTRAL (<http://www.cochrane.org/>)

**Cost-Effectiveness Analysis Registry (CEA)** (http://www.tufts-nemc.org/cearegistry/)

Database of Promoting Health Effectiveness Reviews (DoPHER) (Eppi.ioe.ac.uk/webdatabases/Intro.aspx?ID=2)

**EconLit** (<http://www.econlit.org/>)

Embase ([www.elsevier.com/online-tools/embase](http://www.elsevier.com/online-tools/embase))

Global Health Public Health Database (www.ebscohost.com/academic/global-health)

**Health Economic Evaluations Database (HEED)**
(http://www3.interscience.wiley.com/cgi-bin/mrwhome/114130635/HOME)

Healthcare Management Information Consortium (HMIC) database ([www.ovid.com/site/catalog/DataBase/99.jsp?top=2&mid=3&bottom=7&subsetion=10](http://www.ovid.com/site/catalog/DataBase/99.jsp?top=2&mid=3&bottom=7&subsetion=10))

Index Medicus for the South-East Asia Region (IMSEAR) (library.searo.who.int/modules.php?op=modload&name=websis&file=imsear)

IndMED (India) (indmed.nic.in)

Informit ([www.informit.com.au/health.html](http://www.informit.com.au/health.html))

Intute ([www.intute.ac.uk/](http://www.intute.ac.uk/))

KoreaMed ([www.koreamed.org/SearchBasic.php](http://www.koreamed.org/SearchBasic.php))

Medline (www.ncbi.nlm.nih.gov/pubmed)

National Technical Information Service (NTIS) ([www.ntis.gov](http://www.ntis.gov))

**NHS Economic Evaluation Database (NHS EED)** (<http://www.crd.york.ac.uk/crdweb/>)

PsychInfo (www.apa.org/pubs/databases/psycinfo/)

Scopus (www.scopus.com)

Social policy and practice ([www.ovid.com/site/catalog/DataBase/1859.pdf](http://www.ovid.com/site/catalog/DataBase/1859.pdf))

Turning Research into Practice (TRIP) database ([www.tripdatabase.com/](http://www.tripdatabase.com/index.html))

Western Pacific Region Index Medicus (WPRIM) (wprim.wpro.who.int/SearchBasic.php)

SEARCH STRATEGY

Asia OR “Asia Southeastern” OR “Eastern Asia” OR Oceania OR “Asia Pacific” OR “Pacific Islands” OR “Micronesia” OR “Asia” OR “East Asia” OR “South Asia” OR “Western Pacific” OR “Pacific” OR Cambodia OR Korea OR Myanmar OR Indonesia OR Kiribati OR Laos OR Micronesia OR Mongolia OR Papua New Guinea OR PNG OR Philippines OR Samoa OR Solomon Islands OR Timor-Leste OR Timor Leste OR East Timor OR Vanuatu OR Vietnam OR Viet Nam OR Samoa OR China OR Fiji OR Malaysia OR Marshall OR Palau OR Thailand OR Tonga OR Tuvalu OR Bangladesh OR Nepal OR Bhutan OR India OR Pakistan OR Sri Lanka OR Maldives OR Nauru OR Niue OR Australia OR New Zealand OR Singapore OR Brunei Darussalam OR Japan

AND

“Cardiovascular Diseases” OR “Coronary Artery Disease” OR “Atherosclerosis” OR “Coronary Disease” OR “Myocardial Infarction” OR “Myocardial Ischemia” OR “Stroke” OR “Ischaemic heart disease” OR myocardial OR ischaemic heart disease OR stroke OR brain vascular accident OR cerebrovascular OR cerebrovascular accident* OR CVA OR CVD OR CHD OR cardiovascular disease OR heart disease OR coronary heart disease OR “chronic heart disease” OR cardiovascular OR “cardiovascular event” OR “CVD event”

AND

“Primary prevention” OR ”lifestyle intervention” OR “behaviour* intervention” OR prevention OR taxation OR advertising OR social marketing OR “advertising as topic” OR diet* OR “health promotion”) AND (modification* OR salt OR sodium OR NaCl OR salt reduction OR smoking intervention* OR exercise intervention* OR physical activity OR physical inactivity OR diet OR multiple lifestyle OR dietary intervention* OR diet OR food OR brief advice OR counselling OR incentive based OR active and passive OR home health education OR lifestyle OR incentive based OR “prevention and control” OR “self efficacy” OR “self-efficacy” OR “Counselling” OR “Directive Counselling” OR “Disease Management” OR Behaviour OR “Patient Education as Topic” OR “Sodium Chloride, dietary” OR “trans fats” OR exercise

AND

“Economics” OR “socioeconomic factors” OR “costs and cost analysis” OR “Cost benefit analysis” OR cost effectiveness OR cost utility OR finance* OR economic OR monetary OR cost* OR CUA OR “cost utility analysis” OR cost-outcome OR cost-description OR “cost-consequences analysis”
