# Supplementary material for: Evidence for cost-effectiveness of lifestyle primary preventions for cardiovascular disease in the Asia-Pacific Region: a systematic review
Source: Global Health. 2014 Nov 19;10:79. doi: 10.1186/s12992-014-0079-3 (PMC4251847; doi:10.1186/s12992-014-0079-3)
Supplement: Additional file 2: — Eligibility checklist. [file 12992_2014_79_MOESM2_ESM.docx]

**Additional File 2**

**ELIGIBILITY CHECKLIST**

1. **Study Design**

RCT □ Case-control □ Cohort □ Cross-sectional

Simulation □ Based on:- RCT □ Case-control □ Cohort □ Cross-sectional □

Other…………………………..

Notes ………………………………………………………………………………………………………………………………………………

…………………………………………………………………………………………………………………………………………………….

1. **Participants**

| Criteria | Yes | No | Unclear |
| --- | --- | --- | --- |
| Adults (18yrs+) |  |  |  |
| Located in Asia Pacific Region |  |  |  |

Notes ………………………………………………………………………………………………………………………………………………

…………………………………………………………………………………………………………………………………………………….

1. Interventions

|  | Yes | No | Unclear |
| --- | --- | --- | --- |
| Cardiovascular disease is outcome measure |  |  |  |
| Intervention is lifestyle/behavioural |  |  |  |
| Intervention is primary prevention |  |  |  |
| Control/other intervention is used as comparator |  |  |  |

Notes ………………………………………………………………………………………………………………………………………………

…………………………………………………………………………………………………………………………………………………….

1. Outcomes

|  | Yes | No | Unclear |
| --- | --- | --- | --- |
| Cost-Utility Analysis |  |  |  |
| Cost-effectiveness Analysis |  |  |  |
| Cost-benefit Analysis |  |  |  |

Notes ………………………………………………………………………………………………………………………………………………

…………………………………………………………………………………………………………………………………………
