# Supplementary material for: Evidence for cost-effectiveness of lifestyle primary preventions for cardiovascular disease in the Asia-Pacific Region: a systematic review
Source: Global Health. 2014 Nov 19;10:79. doi: 10.1186/s12992-014-0079-3 (PMC4251847; doi:10.1186/s12992-014-0079-3)
Supplement: Additional file 3: — Data extraction and management. [file 12992_2014_79_MOESM3_ESM.docx]

**Additional File 3**

**Data extraction and management**

| SOURCE | | | | | |  | | | | |
| --- | --- | --- | --- | --- | --- | --- | --- | --- | --- | --- |
| YEAR PUBLISHED | | | | | |  | | | | |
| VOLUME | | | | | |  | | | | |
| STUDY IDENTIFICATION (EG ACCESSION NUMBER) | | | | | |  | | | | |
| COUNTRY OF STUDY | | | | | |  | | | | |
| IMPLEMENTING BODY (OF HEALTH PROMOTION STRATEGY) | | | | | |  | | | | |
| LANGUAGE OF STUDY | | | | | |  | | | | |
| STUDY TYPE | | | | | |  | | | | |
| ECONOMIC STUDY TYPE | | | | | |  | | | | |
| IF SIMULATION, BASIS FOR STUDY (e.g. RCT) | | | | | |  | | | | |
| IF SIMULATION, TYPE and REPETITIONS (MODELLING AND STATISTICAL EXTRAPOLATION INFO) | | | | | |  | | | | |
| IF SIMULATION, CYCLE PERIOD | | | | | |  | | | | |
| LOCATION OF INTERVENTION/SETTING | | | | | |  | | | | |
| YEAR OF STUDY (DATES) | | | | | |  | | | | |
| DURATION OF STUDY | | | | | |  | | | | |
| PARTICIPANT NUMBER | | | | INTERVENTION | |  | | | | |
|  |  |  |  | CONTROL | |  | | | | |
|  |  |  |  | TOTAL | |  | | | | |
| PARTICIPANT DEMOGRAPHY (AGE, SEX, ETHNICITY) | | | | | |  | | | | |
| PARTICIPANT STRATIFICATION (AGE, SEX) | | | | | |  | | | | |
| PARTICIPANT RISK LEVEL (of CVD) | | | | | |  | | | | |
| DESCRIPTION OF INTERVENTION | | |  | | | | | | | |
| DESCRIPTION OF CONTROL | | |  | | | | | | | |
| LINK BETWEEN EFFECTIVENESS AND COST DATA | | | | | |  | | | | |
| MODE OF INTERVENTION PROVISION | | | | | |  | | | | |
| DURATION OF INTERVENTION | | | | | |  | | | | |
| CONTROL/COMPARATOR | | | | | |  | | | | |
| DISCOUNT RATE | | | | | |  | | | | |
| COSTS PERSPECTIVE (HEALTH SYSTEM, SOCIETAL etc.) | | | | | |  | | | | |
| EFFECTIVNESS OF INTERVENTION: CLINICAL EVENTS (MYOCARDIAL INFARCTION, STROKE ETC.) | | | | | |  | | | | |
| MEAN TIME POINT OF CLINICAL EVENTS | | | | | |  | | | | |
| SURVIVAL (MORTALITY RATE) | | | | | | | | | | |
| INTERVENTION | | | | | | CONTROL | | | | |
| MEAN | SD | | | SE | N | MEAN | SD | | SE | N |
|  |  | | |  |  |  |  | |  |  |
| COST-EFFECTIVENESS | | | | | | | | | | |
| TIME POINT | | | | | |  | | | | |
| COST-EFFECTIVENESS INTERVENTION/CONTROL | | | | COST/QALY | |  | |  | | |
|  |  |  |  | COST/DALY | |  | |  | | |
|  |  |  |  | COST/LYS | |  | |  | | |
|  |  |  |  | OTHER | |  | |  | | |
| ICER | | | | COST/QALY | |  | | | | |
|  |  |  |  | COST/DALY | |  | | | | |
|  |  |  |  | COST/LYS | |  | | | | |
|  |  |  |  | OTHER | |  | | | | |
| OTHER INFORMATION | |  | | | | | | | | |
| QUESTIONS ABOUT DATA | |  | | | | | | | | |
| IMPLICATIONS ABOUT STUDY | |  | | | | | | | | |
| CONFLICTS OF INTEREST | |  | | | | | | | | |
