# Supplementary material for: Evidence for cost-effectiveness of lifestyle primary preventions for cardiovascular disease in the Asia-Pacific Region: a systematic review
Source: Global Health. 2014 Nov 19;10:79. doi: 10.1186/s12992-014-0079-3 (PMC4251847; doi:10.1186/s12992-014-0079-3)
Supplement: Additional file 4: — Assessment of risk of bias in included studies. [file 12992_2014_79_MOESM4_ESM.docx]

**Additional File 4**

**Assessment of risk of bias in included studies**

|  | Yes | No | Not Clear | Not Appropriate |
| --- | --- | --- | --- | --- |
| EVERS 2005 |  |  |  |  |
| Is the study population clearly described? |  |  |  |  |
| Are competing alternatives clearly described? |  |  |  |  |
| Is a well-defined research question posed in answerable form? |  |  |  |  |
| Is the economic study design appropriate to the stated objective? |  |  |  |  |
| Is the chosen time horizon appropriate to include relevant costs and consequences? |  |  |  |  |
| Is the actual perspective chosen appropriate? |  |  |  |  |
| Are all important and relevant costs for each alternative identified? |  |  |  |  |
| Are all costs measured appropriately in physical units? |  |  |  |  |
| Are costs valued appropriately? |  |  |  |  |
| Are all important and relevant outcomes for each alternative identified? |  |  |  |  |
| Are all outcomes measured appropriately? |  |  |  |  |
| Are outcomes valued appropriately? |  |  |  |  |
| Is an incremental analysis of costs and outcomes of alternatives performed? |  |  |  |  |
| Are all future cots and outcomes discounted appropriately? |  |  |  |  |
| Are all important variables, whose values are uncertain, appropriately subjected to sensitivity analysis? |  |  |  |  |
| Do the conclusions follow from the data reported? |  |  |  |  |
| Does the study discuss the generalizability of the results to other settings and patient/client groups? |  |  |  |  |
| Does the article indicate that there is no potential conflict of interest of study researcher(s) and funder(s)? |  |  |  |  |
| Are ethical and distributional issues discussed appropriately? |  |  |  |  |
